# Oral and Maxillofacial Infections—A Bacterial and Clinical Cross-Section

**DOI:** 10.3390/jcm11102731

**Published:** 2022-05-12

**Authors:** Bartłomiej Kamiński, Katarzyna Błochowiak, Konrad Kołomański, Maciej Sikora, Sławomir Karwan, Dariusz Chlubek

**Affiliations:** 1 Department of Otolaryngology, Maria Skłodowska-Curie District Hospital, 26-110 Skarżysko-Kamienna, Poland; bartl.kaminski@gmail.com; 2 Department of Oral Surgery and Periodontology, Poznan University of Medical Sciences, 61-701 Poznan, Poland; kblochowiak@ump.edu.pl; 3 Department of Maxillofacial Surgery, Hospital of the Ministry of Interior, 25-375 Kielce, Poland; kolomanskikonrad@gmail.com (K.K.); sikora-maciej@wp.pl (M.S.); 4 Department of Biochemistry and Medical Chemistry, Pomeranian Medical University, 70-111 Szczecin, Poland; 5 Department of Maxillofacial Surgery, Regional Specialized Children’s Hospital, 10-561 Olsztyn, Poland; slawek.karwan@gmail.com

**Keywords:** oral and maxillofacial infections, odontogenic infections, peritonsillar abscesses, deep neck infections, antibiotics, bacteria, oral cavity, maxillofacial areas

## Abstract

The treatment of oral and maxillofacial infections is based on a recognized algorithm that may require modification under the influence of various local and systemic factors. The aim of this study was to present a comprehensive and microbiological profile of oral and maxillofacial infections, and explore possible correlations between the course of an infection and selected systemic factors based on the medical records of 329 patients affected by the disease. We identified most common clinical, demographic, bacterial, and laboratory parameters specific for these infections. There were statistically significant differences in Erythrocyte Sedimentation Rate, number of accompanying diseases, otalgia, dyspnea, and speech difficulties occurrence and neck space involvement between diabetic and non-diabetic patients. The duration of hospitalization and accompanying diseases correlated positively with the patient age and white blood cell count, and C-reactive protein value negatively correlated with age. The primary cause of infections, age, and comorbid diseases can modify the infection course and increase the risk of developing serious complications. It confirms the need for effective and targeted bacterial treatment in the early stages of infections. Age and general diseases are the most important systemic factors determining the infection symptoms and laboratory parameters assessing the severity of the inflammatory process.

## 1. Introduction

The oral cavity and adjacent maxillofacial areas form one of the most diverse spectra of microorganisms in the human body. They are characterized by the presence of saprophytic bacteria that, under favorable conditions, can cause severe infections. In addition, the throat, which crosses the alimentary and respiratory tracts, is prone to infections. Damage to the physiological epithelial defense barriers resulting from microinjuries associated with chewing, daily activities, habits, dental treatment, and wearing of removable dentures predisposes to bacterial infections. Moreover, common odontogenic and maxillofacial infections, including periodontitis, endodontic infections, suppuration, sinusitis, and tonsillitis, can spread to adjacent fascial areas, resulting in life-threatening infections. Although, recently, the incidence of deep neck infections has been decreased with the use of antibiotics; however, they can still lead to lethal complications, such as airway obstruction, Ludwig’s angina, diffuse inflammatory abscess processes, necrotizing fasciitis, purulent meningitis, cerebrospinal abscesses, mediastinitis, sepsis, and septic shock. The treatment of oral and maxillofacial infections is based on surgical treatment involving incision and drainage of the affected spaces. The severity of their course, the spread of infection, and the associated risk of life-threatening complications require additional broad-spectrum antibiotic therapy. Although our diagnostic and therapeutic management is determined by well-known algorithms, many factors accompanying the infection may have a modifying effect on its course and development, forcing changes in the way we deal with these infections [1,2,3,4,5,6,7,8,9]. It seems justified to indicate the most important features characterizing maxillofacial infections for the creation of their clinical and microbiological profile. Despite the progress made in recent years in the diagnosing and treatment of maxillofacial infections, more data are needed to better elucidate the relationship between infections and accompanying systemic diseases and age, and to identify possible causes of infections and potential predictive factors for their more aggressive course. The aim of this study was to present a comprehensive and microbiological profile of oral and maxillofacial infections, and explore possible correlations between the course of an infection and selected systemic factors based on the medical records of 329 patients affected by the disease.

## 2. Materials and Methods

We reviewed the medical records of 329 patients with a diagnosis of oral and maxillofacial infections treated in the Department of Otolaryngology, the Hospital in Skarzysko-Kamienna, and in the Department of Maxillofacial Surgery of Hospital of the Ministry of Interior in Kielce from January 2014 to December 2021. Recorded data included demographic data, etiology, symptoms of infection, site of infection, inflammatory variables, treatment and hospital care details, duration of hospital stay, complications, identified bacterial species, accompanying systemic diseases, and antibiotics used to fight infections. Exclusion criteria included head and neck tumors, superficial skin abscesses, and incomplete patient data. The diagnosis was made on the basis of the history of the patient, clinical examination, and ultrasonic examination or computed tomography (CT) scans imaging. Routine blood tests and bacteriological examinations were carried out. Laboratory tests included Erythrocyte Sedimentation Rate (ESR), white blood cell (WBC) count, and C-reactive protein (CRP) value. The reference ranges for standard values at our laboratory were 4 × 10^3^–10 × 10^3^/mm^3^ for the white blood cell count (WBC), less than 5 mg/L for the c-reactive protein, and 1–10 mm/h for ESR. All patients were treated surgically or in combination with antibiotics. After admission, empirical antimicrobial therapy was implemented as the first choice, and this treatment in selected cases was modified after the results of microbiological analysis. All material for microbiological analysis was collected during surgery and drainage. Both aerobic and anaerobic bacterial cultures were performed using an aseptic technique. Surgical treatment included incision and drainage of abscesses under local or general anesthesia. Moreover, biopsy was performed before surgical incision in selected patients. 

The protocol for this study was approved by the Bioethics Committee of Holycross Medical Chamber, Poland (No 11/2021-VIII). This study was performed in accordance with the ethical standards laid down in an appropriate version of the World Medical Association Declaration of Helsinki. Written informed consent was obtained from each patient before any study procedure was carried out. 

### Statistical Analysis

The calculations were carried out with Microsoft Excel 2016 and STATISTICA software (v.13 TIBCO, Palo Alto, CA, USA). Distributions of continuous variables were evaluated for normality using the Shapiro–Wilk test. The differences between the two groups were tested using the Mann–Whitney U test. Categorical variables are presented in contingency tables, and their associations were tested, depending on the number of cases, with Fisher’s exact test or Chi^2^ Pearson’s test. For qualitative variables, the numbers (*n*) and proportions (%) were calculated and collected in cross-tables. Spearman’s rank correlation analysis was used to find the associations between age and selected clinical and laboratory parameters. *p* < 0.05 was considered statistically significant.

## 3. Results

The summarized patient demographic, clinical, and laboratory characteristics are presented in Table 1. A detailed analysis of the patients’ ages showed that 10% were under 20 years, 48.3% were 21–40 years, 22.7% were 41–60 years, and 17.9% were >60 years. Predominant symptoms in oral and maxillofacial infection were pain (91.9%), followed by dysphagia (48.6%), neck swelling (41.9%), and trismus (39.2%). Most patients reported up to two symptoms of infection. The most frequently occupied areas were the peritonsillar (46.2%), submandibular (27.96%), and buccal (16.41%). CRP value was elevated in 318 patients (96.65%), and ESR value was elevated in 134 patients (40.7%). Leukocytosis was detected in 199 patients (60.48%). The most common cause of infection was tonsilitis (47.1%), followed by odontogenic source (32.2%).

The summarized patient data related to the implemented treatment, duration of hospitalization, identified bacterial species, and detected complications are presented in Table 2. All patients (100%) were treated surgically by incision and drainage. Most procedures (67.1%) were performed under local anesthesia. Surgical treatment was combined with antibiotics in 327 cases. In 252 patients, two synergistic antibiotics were applied. In 16 patients, three antibiotics were applied. A detailed distribution of antibiotics in mono- and polytherapy is presented in Table 2. In 58.05% of the patients, a positive microbiological culture was obtained. Single bacterial strains were isolated in 152 patients, accounting for 79.58%. Multi-bacterial infections were detected in 39 cases, accounting for 20.41%. The most frequently isolated aerobic strains were *Streptococcus mitis* and *Staphylococcus aureus*, whereas the most common anaerobic strains were *Escherichia coli*. Aerobic bacteria were found in 72.25% of the patients, whereas anaerobic bacteria were found in 27.74%. A detailed distribution of cultured bacterial species is presented in Table 2.

### 3.1. Comparison of Clinical Profile between Diabetic and Non-Diabetic Patients

The prevalence of otalgia, dyspnea, and difficulty speaking in diabetic patients was significantly higher than those of non-diabetic patients (*p* = 0.0187, *p* = 0.0002, *p* = 0.0028, respectively). The value of ESR was significantly higher in diabetic patients compared to non-diabetic patients (*p* = 0.0292). There were no differences in CRP and WBC values between diabetic and non-diabetic patients. Diabetic patients presented significantly higher BMI values (*p* = 0.0198) and numbers of accompanying diseases (*p* = 0.0000) compared to non-diabetic patients. Tonsilitis was identified as a cause of maxillofacial infections significantly frequent in diabetic patients (*p* = 0.0125). There was a difference in the cervical space involvement between diabetic and non-diabetic patients (*p* = 0.0064).

What needs to be addressed is that the difference in numbers between the two examined groups of patients is significant (approx. 9.5% were suffering from diabetes), and, therefore, it might raise some concern as to whether this group is statistically valid. According to the WHO data for the year 2021, around 10.5% of the global adult population is suffering from diabetes, which does not diverge from the number of diabetic patients that were included in our study. The parameters that are especially noteworthy are the value of ESR and BMI, which, even on a smaller group of patients, show a difference large enough that it is, in fact, statistically important and not a mere coincidence. The presence of chronic diseases such as diabetes cannot be omitted during any medical examination or study due to the fact that they might greatly influence the individual’s recovery process and prognosis, and, therefore, the result of the study itself. A detailed comparison of selected clinical and laboratory data between the diabetic and the non-diabetic patients is presented in Table 3.

### 3.2. Correlations between Age and Selected Laboratory and Clinical Parameters in Patients with Oral and Maxillofacial Infections

**Figure 1:**
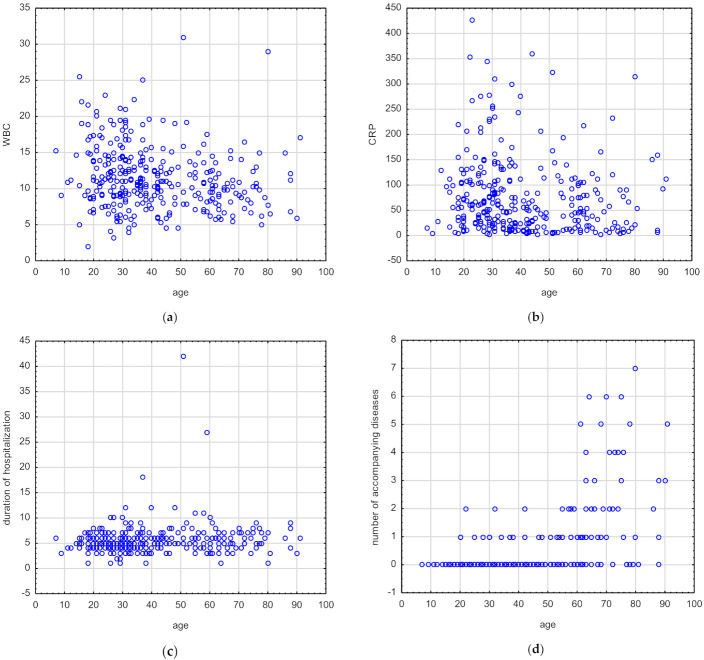
(**a**–**d**) Correlation of WBC, CRP, duration of hospitalization, number of accompanying diseases, and the patient age. The strength of the correlations was determined using Spearman’s rank correlation coefficient; *p* < 0.05 was considered statistically significant.

ESR and number of symptoms did not correlate with age (Table 4). BMI (r_s_ = 0.46, *p* = 0.00), duration of hospitalization (r_s_ = 0.13, *p* = 0.01), and number of accompanying diseases (r_s_ = 0.51, *p* = 0.00) correlated positively with the patient age. In turn, WBC (r_s_ = −0.21, *p* ˂ 0.001) and CRP (r_s_ = −0.16, *p* ≤ 0.001) values negatively correlated with the patient age (Table 4) (Figure 1a–d, respectively).

There were statistically significant differences between age and selected symptoms of infections in patient groups with and without trismus, dysphagia, and dyspnea (*p* = 0.0006, *p* = 0.0178, *p* = 0.0070, respectively), as it is presented in Table 5.

### 3.3. Correlations between Accompanying Diseases and Selected Laboratory and Clinical Parameters in Patients with Oral and Maxillofacial Infections

BMI positively correlated with accompanying diseases (r_s_ = 0.23, *p* = 0.000) (Table 6). WBC value negatively correlated with accompanying diseases (r_s =_ −0.14, *p* = 0.008) (Table 6). CRP, ESR, and duration of hospitalization did not correlate with number of accompanying diseases.

## 4. Discussion

Despite the well-known etiology and clinical course of oral and maxillofacial infections, the exact nature of the relationship between these infections and other factors modifying their course remain to be fully elucidated. In the present study, we made a comprehensive analysis of oral and maxillofacial infections. We also compared the course of these infections in diabetic and non-diabetic patients to indicate most important clinical features in diabetic patients that could modify their course and influence their severity. Likewise, we were able to find a correlation between age and selected clinical and laboratory parameters, as well as between accompanying diseases and clinical features of these infections. Our findings indicated that oral and maxillofacial infections are characterized by specific clinical, laboratory, and demographic profiles that could be helpful in preparing a policy against these infections. Moreover, we revealed a few clinical features with potential for the more severe course of these infections.

In our study, pharyngotonsillar infections were the most common cause of maxillofacial infections. This result is consistent with some previous studies [10,11,12,13]. The second most common identified cause of oral and maxillofacial infections was odontogenic infections. Both causes are most often and interchangeably indicated as the source of oral and maxillofacial infections. The differences in the frequency of the indicated causes of oral and maxillofacial infections appearing in the studies result from differences in the age and ethnic structure of the studied groups, from the different level and availability of dental care, and the state of oral hygiene. Moreover, most odontogenic infections develop as intraoral vestibular abscesses, and rarely progress to more severe deep neck infections. They can be treated by routine incision and drainage without pharmacological support. Currently, early extraction of the causative affected tooth, or its effective endodontic treatment, allow to fight avoid odontogenic infection. Furthermore, malpractice associated with unjustified use of antibiotics in combating odontogenic infections instead of routine surgical and causal treatment can significantly reduce the share of odontogenic infections as the main cause of oral and maxillofacial infections. Good oral hygiene and easy access to dental care may decrease the incidence of odontogenic infections. Another potential reason for the high share of the odontogenic factor in the incidence of maxillofacial infections is the presence of tooth pathologies characteristic for specific age groups. The younger the patient, the more frequently the cause of the infection remains unidentified. This feature of maxillofacial infections was confirmed in study conducted by Yang et al., who pointed to the branchial cleft cyst and thyroglossal duct cyst as more common causes of these infections in children than in adults [13]. In turn, young adults suffer from acute periodontitis, associated with the difficult eruption of the lower wisdom tooth, which is common in this age group, and often indicated as a cause of endodontic infection. In our opinion, a large proportion of the young adults, but not children, in the study group may translate into a statistically higher frequency of odontogenic causes.

The results obtained in our study on demographic data and their distribution depending on gender and age are similar to previous studies [6,12,13,14]. In accordance with other studies, our research confirmed that oral and maxillofacial infections occur most frequently in the age group 21–40 [6]. Male predominance and relatively young age of patients were consistent with previous studies [6,9]. These findings may be a result of a greater exposure to the primary causes of infection in this age group, a higher proportion of odontogenic causes, exposure to potentially damaging factors in the oral cavity, and worse access to dental treatment in the male group. The duration of hospital stay reported in our study was similar to those presented previously [9]. The most common space involvement presented in our study reflects the primary cause of infection, and was closely related to the symptoms reported by patients, such as trismus, dysphagia, and neck swelling. Parapharyngeal and peritonsillar abscess as the most common space involvement was also reported in previous studies [10]. Most of the odontogenic infections indicate the submandibular space as the most common location of these infections [9]. Pain seems to be a common symptom in maxillofacial infections, independent of other clinical features [10]. In turn, the lower share of fever as a symptom of infection in our study may be the result of the small proportion of children in the research group, as previous studies indicated that fever is more common in children than in adults [13]. Some patients presented elevated laboratory parameters related to infection, such as ESR, CRP, and WBC, confirming their important role in the diagnostic process of these infections [10]. Among the assessed parameters, CRP turned out to be the most sensitive indicator for oral and maxillofacial infections confirmed in our study. On the other hand, the traditional laboratory parameters of inflammation detected in our study do not always reflect the severity of oral and maxillofacial infections. Although ESR and WBC help to define the state of the patient on admission, their predictive value is limited. For these infections, the role of other laboratory parameters is emphasized. It is postulated that neutrophil-lymphocyte ratio and IL-6 are important predictors of the number of complications and the number of involved organs in oral and maxillofacial infections [15]. Our results may confirm previous findings that among laboratory parameters of infection, serum CRP value on admission correlates with its severity, and may predict the duration of hospital stay [16]. Moreover, it seems that CRP is a good laboratory parameter for the assessment of oral and maxillofacial infections in patients with accompanying systemic diseases. Its value is not dependent on the number of accompanying diseases, contrary to WBC values, which negatively correlated with accompanying diseases. CRP value is not dependent on diabetes, contrary to ESR. Taken together, it seems that CRP is the most useful and reliable laboratory parameter for the assessment of oral and maxillofacial infections in different patient groups. It is the most sensitive as well as independent on the number and nature of accompanying diseases. Other clinical features of oral and maxillofacial infections include symptoms and occupied fascial areas. Maxillofacial infections are characterized by symptomatology limited to three symptoms of the disease. The type and severity of symptoms and the number of space involvement are more important for the course of the infection than the number of symptoms. Another postulated factor influencing and modifying the profile of the maxillofacial infections is accompanying systemic diseases. The traditional impact of diabetes as a factor of increased risk of maxillofacial infections has been widely discussed in previous studies [17,18]. According to Seppänen et al., in recent years, the incidence of oral and maxillofacial infections has been systematically increasing among compromised patients, from 65% to 83% [19]. In our study, vascular diseases and circulatory disorders, both peripheral and cerebral, have a particularly large share. Moreover, the proportion of patients with cardiovascular disorders and hypertension in maxillofacial infections systematically increased in recent years [19]. Circulatory disorders may contribute to a greater incidence of oral and maxillofacial infections and their more severe course. Similar relationships are observed in the case of odontogenic infections and osteomyelitis, where local blood supply failure is the major cause of the infection spread. Liver and renal failures reported in our study are other potential factors of increased risk of deep neck infections development. Similar results were obtained by Yang et al., who reported that immunocompromised patients, including renal patients with uremia or chronic renal insufficiency and liver patients with cirrhosis, as well as patients with acute myeloid leukemia, presented a longer hospital stay and elevated risk of multispace infections [13]. According to Weise et al., long-term diabetes mellitus, obesity, chronic alcohol abuse, hepatitis, liver cirrhosis, immunosuppression after organ transplantation, chemotherapy, radiotherapy, and systemic lupus erythematosus are predisposing factors for severe odontogenic infections with septic progression [14]. Moreover, medically compromised patients have been known to be at risk for oral infections occurring as common causes of oral and maxillofacial infections [20]. In addition, the mutual relationship between health status and the risk of odontogenic infections is evidenced by the improvement in general condition as a result of improved oral health and oral hygiene [20]. Similar relationships exist between increased BMI and obesity leading to the development of many systemic diseases, and an increased risk of odontogenic infections. These multifactorial associations between oral infections and systemic diseases and mortality are based on immunological and unknown mechanisms, inflammation, and metabolic pathways [20]. Another factor potentially modifying the course of maxillofacial infections is steroid therapy. In our opinion, steroids used in some diseases, such as connective tissue diseases, could be considered as a factor of elevated risk of deep neck infections.

The predominant bacterial species cultured in our study were *Streptococcus mitis Staphylococcus aureus*, followed by *Staphylococcus aureus*, and *Staphylococcus epidermidis*. Previous studies identified various pathogens as a major cause of oral and maxillofacial infections [12,21]. These discrepancies resulted from the impact of other modifying factors. Tsai et al. revealed a relationship between the identified bacterial species and the patient age and obesity. Obesity, described as a BMI value above 27, was associated with a higher isolation of *Peptostreptococcus*. Furthermore, elderly patients above 65 years old had higher *Klebsiells pneumoniae* isolation [12]. Contrary to other studies, the same authors did not detect any associations between diabetes and isolated pathogens [12]. In turn, Celakovsky et al. reported that the most common aerobic organisms playing a causative role in deep neck infections are *Streptococcus viridans*, β-haemolytic *Streptococcus*, *Staphylococcus aureus*, and *Klebsiella pneumonie.* In the same study, the incidence of anaerobic bacteria was higher in adults, in patients with infections of dental origin, and in non-diabetic patients [21]. In general, most of the studies reported Gram-positive cocci as the most common aerobic pathogen in oral and maxillofacial infections. Moreover, in odontogenic infections, the predominant cultured bacterial species was *Streptococcus viridians* [12,14,22,23]. These findings are consistent with our results. These bacterial species are common oral flora normally found in the mouth, and they could be causative pathogens for odontogenic infections. These differences in the contribution of selected bacteria to maxillofacial infections may be due to the different age structure, comorbidities, and causes of infection, and even oral hygiene and lifestyle [12]. Odontogenic infections are characterized by a greater proportion of anaerobic bacteria and their polymicrobial nature. In turn, a large share of pharyngitis and tonsillitis as a cause of maxillofacial infections may be associated with the predominance of streptococci. According to Galioto et al., group A streptococcus and the *Streptococcus milleri* group, a subgroup of viridans streptococci, including *S. intermedius*, *S. anginosus*, and *S. constellatus*, are the most commonly isolated aerobes in peritonsillar abscess [24,25]. These finding reflect a significant share of tonsillitis and pharyngitis as causes of oral and maxillofacial infections detected in our study. Although we have isolated aerobic bacterial strains characteristic of peritonsillar abscesses, we have not been able to identify *Fusobacterium necrophorum*, described as the most common anaerobic bacterium for peritonsillar abscesses [26]. This could be due to difficulties in growing anaerobic bacteria. This microbial profile is characteristic for multimicrobial peritonsillar abscess, but in monomicrobial infections, *Streptococcus pyogenes* predominates [27]. In turn, a frequently observed principle in odontogentic infection is the high proportion of aerobic bacteria in the initial stage of infection which decreases in favor of anaerobic bacteria in the more advanced stages of infections [28]. Bacterial samples obtained during the initial 1–2 days of clinical symptoms manifesting as cellulitis contain facultative bacteria, mostly streptococci. Anaerobes begin to occur during suppuration and abscess formation. The time of the microbiological examination in relation to the beginning of the infection and its cause have the most important influence on the proportions of the identified bacterial species. In our opinion, a significant share of peritonsillar abscess and pharyngitis and tonsillitis as the cause of infections identified in our study had a decisive influence on the dominance of aerobic bacteria and the monobacterial culturing.

It is postulated that diabetic patients are more susceptible to bacterial infections, leading to significant morbidity and mortality. They present dysfunction in polymorphonuclear and neutrophil bactericidal functions, cellular immunity, and complement activation. Therefore, they are prone to a higher incidence and severity of maxillofacial infections, longer duration of hospitalization, and higher rates of complications compared to healthy individuals [17,18]. Although the aim of our study was not to compare maxillofacial infections in diabetic and non-diabetic patients due to the heterogeneity of the size of both groups, it seems that the course and features of oral and maxillofacial infections in people with diabetes show some unique properties and tendencies. These observations prove the need of implementation of other treatments, diagnosis, and surveillance methods in diabetic patients. Diabetic patients more often exhibit dyspnea and speech disorders in comparison to patients without diabetes, which confirms that diabetes may be a significant factor modifying the course of infection and increasing the risk of its severe course. These symptoms may be potentially associated with a higher occurrence of airway obstruction. Our results indicated that diabetes may predispose to tonsilitis, presenting in our study as the most frequent cause of maxillofacial infections in the diabetic group of patients, or that tonsilitis in diabetic patients may develop as deep neck infections more frequently compared to non-diabetic patients. Moreover, according to our results, diabetes predisposes to cervical involvement, characterized as a potentially more severe anatomical site of infection. These findings may justify the need for more careful monitoring and aggressive treatment of pharyngitis and tonsilitis in diabetic patients. Another interesting feature of maxillofacial infections in diabetic patients is the statistically more frequent occurrence of otalgia in the course of these infections. This could be associated with more frequent neurologic impairment in diabetic patients compared to non-diabetic patients. Furthermore, different bacterial species identified in both diabetic and non-diabetic patients and the predominance of aerobic bacteria over anaerobic bacteria in diabetic patients reported in previous studies may explain the differences in the causes of oral and maxillofacial infections and the frequent occurrence of tonsillitis in diabetic patients reported in our study [18,21]. It is postulated that there is a greater share of anaerobic bacteria and a more frequent cause of odontogenic infection in non-diabetic patients compared to diabetic patients [21]. Although the *Streptococcus* species is the most common organism isolated in both diabetic and non-diabetic patients, *Klebsiella pneumoniae* is the second most common organism isolated in diabetic patients, and quite a frequent bacterial species for infections in diabetic patients [17]. Moreover, a large contribution of *Klebsiella pneumoniae* in the maxillofacial infections in diabetic patients is independent of the age [13]. These characteristics of the infection course in diabetic patients are not, however, associated with a statistically significantly increased duration of hospitalization. This contrasts with the other study conducted by Zhang et al., who reported a longer duration of hospitalization in diabetic patients compared to non-diabetic patients [18]. However, length of hospital stay did not correlate with any of the variables that measure the severity of infection. Diabetes alone seems to be an insufficient predictive factor for longer hospitalization. Diabetic patients are prone to two or multi-space infections and more severe complications, such as airway obstruction and descending necrotizing mediastinitis. In a multivariate model for assessing the possible complications in diabetic patients, only admission blood glucose levels and controlling of its level were significantly associated with the complications rate [17,18]. According to Mejzlik et al., the incidence of *Candida albicans* was significantly higher in diabetics than non-diabetics, and was a significant risk factor for life-threatening complications [29]. Moreover, diabetes and older age may be potential risk factors for developing severe infections. These data are consistent with our observation that the risk of complications and of a more severe course of disease increases with age, especially in patients with diabetes mellitus. In our study, older age was associated with multiple morbidity, which may be associated with a more severe course of maxillofacial infections.

Another factor having a potential influence on the course of maxillofacial infections is the patient age. The patient morbidity, BMI, and length of hospitalization increase with age. Similar results were obtained in other studies where patient age positively correlated with length of hospital stay and a higher prevalence of complications [13,30,31,32]. These relationships also result from the concept of the physiological reserve which determines the body’s ability to function under stress and fight infections. With age, the physiological reserves, including metabolic, cardiovascular, and respiratory reserves, decrease. Furthermore, multicomorbidity intensifies the depletion of the physiological reserve, and constitutes an additional factor modifying the course of the infection and increasing the risk of its severe course [1]. Although Yang et al. reported that older age predisposes to multiple infections, in our study, there were no differences in the causes of infection, the number of involved spaces, and location of infection with an increased patient age, which is consistent with previous studies [13]. Moreover, age does not increase the laboratory parameters that usually reflect the severity of infections, such as WBC, CRP, and ESR. On the contrary, CRP and WBC accompanying infection decrease with age. The elder the patient, the lower the CRP and WBC levels. Similar findings were obtained by Yang et al., who reported that mean leucocyte and lymphocyte counts in the children group were both significantly higher than in adults [13]. Several recent studies have highlighted changes in the bacterial load of maxillofacial infections with age [31,33,34]. A relatively higher percentage of the younger patients had negative bacterial culture results in comparison to those in the senior group [31]. Attention is drawn to the greater proportion of anaerobic bacteria in elderly patients. In the study conducted by Slouka et al., the incidence of anaerobic bacteria in the >50 years group was increased compared to other younger groups. The higher the age, the higher the contribution of anaerobes in oral and maxillofacial infections. The higher proportion of the anaerobic spectrum in elderly patients was a result of a higher incidence of metabolic and cardiopulmonary comorbidities in elderly patients. Moreover, the same authors reported the absence of yeast in the 18 years or younger age group [35]. This affects the different course of infections in elderly patients, and may have clinical implications in the need to use other antibiotics [21,36]. One of the possible explanations for these differences is a weaker immune system response in the elderly that progresses with multimorbidity, as well as an accumulative chronic exposure to antibiotic agents, which contributes to antibiotic insensitivity in seniors.

It is worth addressing the fact that our study has been conducted in the years 2020–2021 during the ongoing COVID-19 pandemic. Therefore, we can assume that the progression of diseases, especially the infectious ones, to more advanced stages would have been more common due to limited access to primary medical care. The initial screening is a crucial part of any diagnostic process, as well as early admission of patients to a medical facility. Many hospital wards have been temporarily closed or transformed into COVID-19 wards, which has greatly impacted the accessibility of treatment for non-COVID patients. Additional studies would have to be carried out in order to compare the statistics and present the results in a scientific way and not just speculation.

## 5. Conclusions

Although oral and maxillofacial infections are characterized by a specific clinical, demographic, and microbiological profile, the primary cause of infections, and age and comorbid general diseases can significantly modify their course and increase the risk of developing serious complications. As the aim of this study presented in the beginning was to explore possible correlations between the primary cause of infections and their symptoms and bacterial profile, we confirmed that there is a strong relationship between those agents. This confirms the need for effective and targeted bacterial treatment in the early stages of infections, depending on various factors, such as the existence of chronic diseases or the patient’s age. Both of these systemic factors are the most important when it comes to modifying the course of infections. To the greatest extent, they determine the symptoms of infection and laboratory parameters assessing the severity of the inflammatory process.

## Figures and Tables

**Table 1:** Demographic, clinical, and laboratory characteristics of oral and maxillofacial infection patients.

Parameters	Values
Number of individuals, *n*	329
Gender, Female/Male, *n*	137/192
Age, mean ± SD years	40.88 ± 18.15
Age, median (range), years	36.00 (7.00–91.00)
BMI (kg/m^2^), median	24.02
**Symptoms, ** ***n*** ** (%):**	
Pain	302 (91.8)
Trismus	129 (39.2)
Dysphagia	160 (48.6)
Otalgia	12 (3.6)
Fever	47 (14.2)
Dyspnea	8 (2.4)
Neck swelling	138 (41.9)
Sialorrhea	1 (0.3)
Hoarseness	4 (1.2)
Other symptoms	106 (32.2)
**Number of symptoms, *****n***:	
≤2 symptoms	147
3 symptoms	107
≥4 symptoms	75
**Space involvement, ** ***n*** ** (%)**	
Submandibular	92 (27.96)
Parapharyngeal	8 (2.43)
Peritonsillar	152 (46.2)
Buccal	54 (16.41)
Parotid	5 (1.52)
Temporal	3 (0.60)
Infratemporal	3 (0.60)
Submental	5 (1.52)
Orbital	5 (1.52)
Cervical	20 (6.07)
Lacrimal sac	1 (0.30)
Other space involvement (tongue, lip, epiglottis, lower pharynx)	7 (2.12)
**Laboratory values**	
WBC (K/μL)	10.94
ESR (mm/h)	44.00
CRP (mg/L)	55.48
**Causes, ** ***n*** ** (%)**	
Odontogenic	106 (32.2)
Tonsilitis	155 (47.1)
Sialoadenitis	6 (1.8)
Sinusitis	1 (0.3)
Posttraumatic	13 (3.9)
Postoperative	10 (3.0)
Other/Undetermined	33 (10.0)
**Number of accompanying diseases, *****n***:	
One disease	41
≥Two diseases	26
**Accompanying systemic diseases, *****n***:	
Peripheral vascular diseases	42
Circulatory failure	32
Diabetes without complications	24
Diabetes with complications	7
Cerebrovascular diseases	13
Connective tissue diseases	7
Chronic obstructive pulmonary disease	6
Liver failure	6
Renal failure	5
Dementia	5
Other: Solid tumor, Peptic ulcer disease, Paresis, Stroke, Myocardial infarction, Disseminated tumor	10

SD, standard deviation; *n*, number; %, percentage; BMI, body mass index; WBC, white blood cells; ESR, Erythrocyte Sedimentation Rate; CRP, C-Reactive Protein.

**Table 2:** Detailed data related to hospital stay, implemented treatment, identified bacterial species, and complications.

Parameters	Values
Duration of hospitalization, median (range), days	5.00 (1.00–42.00)
**Antibiotics ** ***n***	
No use of antibiotics	2
Use of single antibiotic	327
First-choice antibiotics: cefuroxime, lincomycin, penicillin, gentamicin, amoxicillin + clavulanic acid, meropenemum, clindamycin, azithromycin, vancomycin	
Multi-antibiotic therapy:	
Use of two antibiotics	
Second choice antibiotics: metronidazole, cefazolin, cefuroxime, clindamycin, gentamycin	
Third choice antibiotics: gentamycin, cefuroxime, metronidazole, penicillin, clindamycin, lincomycin,	252
Use of three antibiotics	
**Microbiology ** ***n***	16
**Gram-positive bacteria:**	
*Staphylococcus:*	
*Aureus*	35
*Epidermidis*	25
*Capitis*	4
*Hominis*	4
Other: *Warneri*, *Auricularis*, *Xylosus*	3, 1, 1
*Streptococcus:*	
*Mitis,*	40
*Anginosus,*	20
*Haemolyticus,*	12
*Sanguinis,*	8
*Mutaris,*	8
*Oralis,*	4
*Identified as gr C*	4
Other: *Salivarius, Parasanguinis, Constellatus, Pluranimalium,*	3, 3, 3, 3
*Intermedius Pseudoporcinus, Agalactiae,*	2, 2, 2
*Ovis, Gordonii, Liquefaciens, Vestibularis*	1, 1, 1, 1
*Enterococcus: faecalis, casseliflavus*	6, 1
*Leuconostoc mesenteroides*	2
*Eggerthella lenta*	1
**Gram-negative bacteria:**	
*Pseudomonas aerigunosa*	11
*Acinetobacter baumannii*	2
*Escherichia coli*	6
*Klebsiella pneumoniae*	5
*Moraxella catarrhalis*	1
*Citrobacter: freundii, brakii*	5
*Enterobacter cloacae*	5
*Serratia marcescens*	2
**Anaerobic bacteria:** *Leuconostoc mesenteroides, Eggerthella lenta,*	
*Escherichia coli*	9
*Candida albicans*	246
Biopsy	
**Type of anesthesia *n*, (%)**	
*Local anesthesia*	221 (67.1)
*General intravenous anesthesia*	5 (1.51)
**Complications *n*, (%)**	
*Tracheostomy*	3 (0.91)
*Intubation*	10 (3.03)

*n*, number; %, percentage.

**Table 3:** Comparison of selected clinical and laboratory data between the diabetic and the non-diabetic patients.

Parameters	Non-Diabetic Patients	Diabetic Patients	*p* Value
Number of individuals, *n*	297	31	
Age, years, mean (±SD)	39.12 (±17.37)	57.77 (±16.98)	
Age, years, median (confidence interval)	35.00 (27.00–48.00)	61.00 (51.00–71.00)	˂0.0001 ^a^
BMI (kg/m^2^), median (confidence interval)	23.79 (21.46–27.27)	27.48 (22.83–31.25)	0.0198 ^a^
WBC (K/μL) median (confidence interval)	11.00 (8.68–13.87)	10.72 (8.90–14.00)	0.5491 ^a^
ESR (mm/h) median (confidence interval)	41.00 (26.00–66.00)	72.50 (32.00–116.50)	0.0292 ^a^
CRP (mg/L) median (confidence interval)	55.61 (23.06–105.44)	44.01 (12.50–114.00)	0.5552 ^a^
Duration of hospitalization (days)	5.00 (4.00–6.00)	6.00 (4.00–7.00)	0.0631 ^a^
Number of accompanying diseases	0.00 (0.00–0.00)	1.00 (0.00–3.00)	0.0000 ^a^
**Symptoms**			
Number of symptoms	3.00 (2.00–3.00)	2.00 (2.00–5.00)	0.8470 ^a^
Pain *n*, yes/no	274/24	28/3	0.7299 ^c^
Trismus, *n*, (%) yes/no	120 (40.27)/178 (59.73)	9 (29.03)/22 (70.97)	0.2226 ^b^
Dysphagia, *n*, (%) yes/no	144 (48.32)/154 (51.68)	16 (51.61)/15 (48.39)	0.7271 ^b^
Otalgia *n*, (%) yes/no	8 (2.68)/290 (97.32)	4 (12.90)/27 (87.10)	0.0187 ^c^
Fever *n*, (%) yes/no	41 (13.76)/257 (86.24)	6 (19.35)/25 (80.65)	0.4174
Dyspnea *n*, (%) yes/no	3 (1.01)/294 (98.99)	5 (16.13)/26 (83.87)	0.0002
Hoarseness *n*, (%) yes/no	1 (0.34)/297 (99.66)	3 (9.68)/28 (90.32)	0.0028 ^c^
Sialorrhea *n*, (%) yes/no	1 (0.34)/297 (99.66)	0 (0.00)/31 (100.00)	1.0000 ^c^
Cervical swelling *n*, (%) yes/no	125 (41.95)/173 (58.05)	13 (41.94)/18 (58.06)	0.9990 ^b^
**Space involvement**			
Submandibular *n*, (%)	82 (27.52)	10 (32.26)	0.5756 ^c^
Buccal *n*, (%)	48 (16.11)	6 (19.35)	0.6422 ^b^
Parotid *n*, (%)	4 (1.34)	1 (3.23)	0.3922 ^c^
Temporal *n*, (%)	1 (0.34)	1 (3.23)	0.1798 ^c^
Infratemporal *n*, (%)	1 (0.34)	1 (3.23)	0.1798 ^c^
Submental *n*, (%)	3 (1.01)	2 (6.45)	0.0718 ^c^
Orbital *n*, (%)	4 (1.34)	1 (3.23)	0.3922 ^c^
Lacrimal sac *n*, (%)	1 (0.34)	0 (0.00)	1.0000 ^c^
Cervical *n*, (%)	14 (4.70)	6 (19.35)	0.0064 ^c^
**Causes**			
Odontogenic *n*, (%)	93 (31.21)	13 (41.94)	0.2238 ^b^
Tonsillitis *n*, (%)	147 (49.33)	8 (25.81)	0.0125 ^b^
Sialoadenitis *n*, (%)	5 (1.68)	1 (3.23)	0.4504 ^c^
Sinusitis *n*, (%)	1 (0.34)	0 (0.00)	1.0000 ^c^
Posttraumatic *n*, (%)	13 (4.36)	0 (0.00)	0.6205 ^c^
Postoperative *n*, (%)	9 (3.02)	1 (3.23)	1.0000 ^c^
Iatrogenic *n*, (%)	2 (0.67)	0 (0.00)	1.0000 ^c^
Unidentified *n*, (%)	4 (1.34)	1 (3.23)	0.3922 ^c^
Complications *n*, (%)	0 (0.00)	1 (3.23)	0.0942 ^c^

SD, standard deviation; *n*, number; BMI, Body mass index; WBC, white blood cells; ESR, Erythrocyte Sedimentation Rate; CRP, C-Reactive Protein; %, percentage; ^a^ Mann–Whitney U test; ^b^ Fisher’s exact test; ^c^ Chi^2^ Pearson’s test.

**Table 4:** Correlations between age and BMI, WBC, CRP, ESR, duration of hospitalization, number of symptoms, and number of accompanying diseases. The strength of the correlations was determined using Spearman’s rank correlation coefficient; *p* < 0.05 was considered statistically significant.

	Parameters	r_s_	*p* Value
Age	BMI	0.46	0.00
	WBC	−0.21	˂0.001
	CRP	−0.16	˂0.001
	ESR	0.07	0.39
	Duration of hospitalization	0.13	0.01
	Number of symptoms	−0.10	0.06
	Number of accompanying diseases	0.51	0.00

BMI, body mass index; WBC, white blood cells; CRP, C-reactive protein; ESR, erythrocyte sedimentation rate.

**Table 5:** Comparison of selected symptoms of infection and age between patients presenting these symptoms and not presenting these symptoms.

Symptoms	Mean ± SD	Median	Min	Max	Q1	Q3	*p*-Value
**Pain**							
Yes, *n* = 301	40.94 ± 18.04	36.00	7.00	91.00	27.00	55.00	0.7013
No, *n* = 27	40.22 ± 19.69	34.00	14.00	81.00	27.00	57.00	
**Trismus**							
Yes, *n* = 128	36.70 ± 16.41	31.00	12.00	91.00	26.00	44.00	0.0006
No, *n* = 200	43.56 ± 18.74	40.00	7.00	90.00	29.00	59.00	
**Dysphagia**							
Yes, *n* = 159	38.60 ± 17.89	33.00	7.00	91.00	26.00	49.00	0.0178
No, *n* = 169	43.02 ± 18.19	38.00	9.00	90.00	29.00	58.00	
**Otalgia**							
Yes, *n* = 12	38.00 ± 17.92	30.00	18.00	80.00	27.50	45.50	0.4959
No, *n* = 316	40.99 ± 18.18	36.00	7.00	91.00	27.00	55.00	
**Fever**							
Yes, *n* = 47	38.44 ± 19.23	31.00	15.00	88.00	25.00	42.00	0.1621
No, *n* = 281	41.29 ± 17.97	36.00	7.00	91.00	28.00	55.00	
**Neck swelling**							
Yes, *n* = 137	40.42 ± 16.76	36.00	18.00	88.00	27.00	52.00	0.8478
No, *n* = 191	41.21 ± 19.12	36.00	7.00	91.00	27.00	58.00	
**Dyspnea**							
Yes, *n* = 8	60.12 ± 19.32	58.50	33.00	86.00	45.00	77.50	0.0070
No, *n* = 319	40.34 ± 17.88	36.00	7.00	91.00	27.00	54.00	

Q1, lower quartile; Q3, upper quartile.

**Table 6:** Correlations between number of accompanying diseases and BMI, WBC, CRP, ESR, and duration of hospitalization. The strength of the correlations was determined using Spearman’s rank correlation coefficient; *p* < 0.05 was considered statistically significant.

	Parameters	r_s_	*p* Value
**Accompanying diseases**	BMI	**0.23**	**0.000**
	WBC	**−0.14**	**0.008**
	CRP	−0.06	0.25
	ESR	0.14	0.07
	Duration of hospitalization	0.07	0.14

BMI, body mass index; WBC, white blood cells; CRP, C-reactive protein; ESR, erythrocyte sedimentation rate.

## Data Availability

Not applicable.
